# *TP53* and *lacZ* mutagenesis induced by 3-nitrobenzanthrone in Xpa-deficient human *TP53* knock-in mouse embryo fibroblasts

**DOI:** 10.1016/j.dnarep.2015.11.004

**Published:** 2016-03

**Authors:** Jill E. Kucab, Edwin P. Zwart, Harry van Steeg, Mirjam Luijten, Heinz H. Schmeiser, David H. Phillips, Volker M. Arlt

**Affiliations:** a Analytical and Environmental Sciences Division, MRC-PHE Centre for Environment & Health, King’s College London, London SE1 9NH, United Kingdom; b Center for Health Protection, National Institute for Public Health and the Environment (RIVM), Bilthoven 3721 MA, The Netherlands; c Division of Radiopharmaceutical Chemistry (E030), German Cancer Research Center (DKFZ), Im Neuenheimer Feld 280, 69120 Heidelberg, Germany

**Keywords:** *TP53*, Mutation, Nucleotide excision repair, 3-Nitrobenzanthrone, DNA adduct, Environmental carcinogen

## Abstract

3-Nitrobenzanthrone (3-NBA) is a highly mutagenic compound and possible human carcinogen found in diesel exhaust. 3-NBA forms bulky DNA adducts following metabolic activation and induces predominantly G:C > T:A transversions in a variety of experimental systems. Here we investigated the influence of nucleotide excision repair (NER) on 3-NBA-induced mutagenesis of the human tumour suppressor gene *TP53* and the reporter gene *lacZ*. To this end we utilised *Xpa* -knockout (Xpa-Null) human *TP53* knock-in (Hupki) embryo fibroblasts (HUFs). As Xpa is essential for NER of bulky DNA adducts, we hypothesized that DNA adducts induced by 3-NBA would persist in the genomes of Xpa-Null cells and lead to an increased frequency of mutation. The HUF immortalisation assay was used to select for cells harbouring *TP53* mutations following mutagen exposure. We found that Xpa-Null Hupki mice and HUFs were more sensitive to 3-NBA treatment than their wild-type (Xpa-WT) counterparts. However, following 3-NBA treatment and immortalisation, a similar frequency of *TP53*-mutant clones arose from Xpa-WT and Xpa-Null HUF cultures. In cells from both *Xpa* genotypes G:C > T:A transversion was the predominant *TP53* mutation type and mutations exhibited bias towards the non-transcribed strand. Thirty-two percent of 3-NBA-induced *TP53* mutations occurred at CpG sites, all of which are hotspots for mutation in smokers’ lung cancer (codons 157, 158, 175, 245, 248, 273, 282). We also examined 3-NBA-induced mutagenesis of an integrated *lacZ* reporter gene in HUFs, where we again observed a similar mutant frequency in Xpa-WT and Xpa-Null cells. Our findings suggest that 3-NBA-DNA adducts may evade removal by global genomic NER; the persistence of 3-NBA adducts in DNA may be an important factor in its mutagenicity.

## Highlights

• We examine *TP53* and *lacZ* mutagenesis induced by 3-nitrobenzanthrone (3-NBA).
• We utilize Hupki (Human *TP53* knock-in) fibroblasts (HUFs) wild-type and null for Xpa.
• Xpa-Null HUFs are more sensitive to 3-NBA than Xpa-WT HUFs.
• 3-NBA induces a similar *TP53* and *lacZ* mutant frequency in Xpa-WT and Xpa-Null HUFs.
• 3-NBA induces mutations at *TP53* codons that are hotspots in smokers’ lung cancer.

## 1 Introduction

Lung cancer is the most common malignant disease worldwide. While tobacco smoking is the predominant cause of lung cancer, vehicular exhaust and ambient air pollution are also implicated [1], [2]. In Great Britain diesel engine exhaust is the sixth most important occupational carcinogen [3]. Moreover, two recent epidemiological studies of miners provide strong evidence for a link between diesel exposure and lung cancer risk, finding a 3-fold increased risk for lung cancer overall and a 5-fold increased risk for miners most heavily exposed to diesel exhaust [4], [5]. Subsequently, the International Agency for Research on Cancer (IARC) has classified diesel engine exhaust as Group 1 human carcinogen [2].

The extremely potent mutagen 3-nitrobenzanthrone (3-nitro-7H-benz[*de*]anthracen-7-one, 3-NBA) can be found on the surface of ambient air particulate matter and diesel exhaust particles [6] and has been classified as a possible human carcinogen (Group 2B) by IARC [2]. Human exposure to 3-NBA has been demonstrated in workers occupationally exposed to diesel emissions [7] and intratracheal instillation of 3-NBA induces squamous cell carcinoma in rat lung [8]. Premutagenic bulky DNA adducts generated by 3-NBA are critical for its high mutagenic potency [9], [10]. In order to form DNA adducts, 3-NBA requires metabolic activation via reduction of its nitro group [11], [12]. The predominant DNA adducts detected *in vitro* and *in vivo* after treatment with 3-NBA are at guanine residues, *i.e.* 2-(2′-deoxyguanosin-*N*^2^-yl)-3-aminobenzanthrone (dG-*N*^2^-3-ABA) and *N*-(2′-deoxyguanosin-8-yl)-3-aminobenzanthrone (dG-C8-*N*-3-ABA) (see Supplementary Fig. 1) [12], [13]. The mutagenic potency of 3-NBA has been shown in a variety of experimental systems [14], [15]. 3-NBA-DNA adducts preferentially induce G:C > T:A transversions *in vitro* and *in vivo* in reporter genes [9], [10], [16].

3-NBA was also shown to induce G:C > T:A transversions in the tumour suppressor gene *TP53* in immortalized embryonic fibroblasts derived from the human *TP53* knock-in (Hupki) mouse, which harbours exons 4–9 of human *TP53* in place of the corresponding mouse exons [17] [18]. *TP53*, which encodes the protein p53, is a key cancer gene and is somatically mutated in approximately half of human cancers [19], [20]. Over 28,000 *TP53* mutations detected in human tumours have been catalogued in the IARC *TP53* mutation database [21]. Some of these mutations in *TP53* bear hallmarks of carcinogen exposure and can offer clues to tumour aetiology [22]. Carcinogen-induced *TP53* mutagenesis can be studied *in vitro* using the Hupki mouse embryo fibroblast (HUF) immortalization assay (HIMA) [23], [24]. HUFs that acquire *TP53* mutations following mutagen treatment can bypass culture-induced senescence and become immortalised clones, in which mutations can be identified by DNA sequencing. The HIMA allows a direct comparison between mutations induced *in vitro* in a human cancer gene (**i.e.* TP53*) and mutations that occur in tumours of populations exposed to a particular carcinogen.

Bulky DNA adducts, such as those formed by polycyclic aromatic hydrocarbons (PAHs), can be removed from the genome by the nucleotide excision repair (NER) pathway [25], [26]. We developed a Hupki mouse strain harbouring a knockout allele for a critical NER component, Xpa (xeroderma pigmentosum complementation group A) (*Hupki*; *Xpa*^+/−^), with the aim of increasing *TP53* mutation frequency in the HIMA [27]. Xpa is critical for both global genomic NER (GG-NER) and transcription-coupled NER (TC-NER), thus *Xpa*^−/−^ (Xpa-Null) HUFs derived from *Hupki*; *Xpa*^+/−^ mice are completely deficient in NER and incapable of removing bulky DNA adducts from their genome [25]. We recently showed that Xpa-Null HUFs, compared with *Xpa*^+/+^ (Xpa-WT) HUFs, are extremely sensitive to the PAH benzo[*a*]pyrene (BaP) and exhibit increased *TP53* mutagenesis on the transcribed strand when treated with the reactive intermediate of BaP, benzo[*a*]pyrene-7,8-diol-9,10-epoxide (BPDE) [27].

In the present study, we examined the impact of NER-deficiency on 3-NBA-induced adduct formation (*in vitro* and *in vivo*) and *TP53* mutagenesis in HUFs. We hypothesized that 3-NBA-induced DNA adducts would persist in the genomes of Xpa-Null Hupki mice and HUFs, and that these persistent adducts would lead to increased mutation induction during replication of adducted DNA [28], [29]. In addition to *TP53* mutagenesis, we examined 3-NBA-induced mutations in a *lacZ* reporter gene in Xpa-WT and Xpa-Null HUFs derived from *Hupki*; *Xpa* ^+/−^ mice, as the pUR288 plasmid locus has also been integrated into this strain [30]. The pUR288 plasmid can be extracted from the genomic DNA of mutagen-treated cells following 1–3 days of proliferation and *lacZ* mutations identified by selection in *Escherichia coli* host cells [31]. In this way, the frequency of mutations induced by carcinogen treatment may be studied in a short-term reporter gene assay (in a manner that is not dependent on selection within HUFs) in parallel to *TP53*. As the HIMA takes several months to complete, we aimed to evaluate whether the mutagenic activity of a carcinogen (*i.e.* 3-NBA) could be rapidly assessed in HUFs using the *lacZ* system. This would potentially aid in the optimisation of experimental treatment conditions prior to initiation of the HIMA.

## 2 Materials and methods

### 2.1 Carcinogen information

3-NBA was synthesised as described previously [32]. For *in vivo* treatments 3-NBA was dissolved in tricaprylin at a concentration of 0.5 mg/mL. For treatment of cells in culture, 3-NBA was dissolved in DMSO to a stock concentration of 4 mM and stored at −20 °C.

### 2.2 Description of mouse strain and details of genotyping

A mouse strain homozygous for the *Hupki* allele (a knock-in allele harbouring exons 4–9 of the human *TP53* gene) and heterozygous for an *Xpa*-knockout allele was generated as described previously [27]. This strain is on a mixed 129/Sv and C57Bl/6 background and also harbours the pUR288 plasmid (containing the bacterial *lacZ* mutation marker gene) [30], [33]. The pUR288 plasmid is chromosomally integrated in ∼20 tandem copies per haploid genome. More information can be found at the European Mouse Mutant Archive (EMMA; www.infrafrontier.eu) where this strain has been deposited (EMMA ID EM:08137). *Hupki*^+/+^; *Xpa*^+/−^; *lacZ* ^+^ mice were bred to produce *Xpa*^+/+^ (Xpa-WT) and *Xpa*^−/−^ (Xpa-Null) mice and embryos for experiments. Genotyping for *Hupki*, *Xpa* and *lacZ* was performed as described previously [27] using the primers and PCR conditions detailed in Supplementary Table 1. Note that genotyping for *lacZ* cannot differentiate between genomes that are haploid or diploid for pUR288 but this does affect the outcome of the *lacZ* mutation assay.

### 2.3 In-vivo 3-NBA treatment

All animal experiments were carried out under license according to protocols approved by the Home Office under the Animals (Scientific Procedures) Act (1986) after approval by the institutional ethics committee. Groups of female Xpa-WT and Xpa-Null Hupki mice (∼3 months old; 20–25 g; *n* = 3/group) were treated with 3-NBA following a treatment protocol published previously [34]. In Groups 1–3 mice were treated with 2 mg/kg bw 3-NBA by intraperitoneal (i.p.) injection. Groups 1 and 2 received a single dose and were sacrificed 1 day and 5 days after the last administration, respectively. Group 3 was dosed once daily for 5 days and sacrificed 24 h after the last injection. Groups 4 and 5 were treated i.p. with 0.2 mg/kg bw 3-NBA. Whereas Group 4 was treated with a single dose and sacrificed 24 h later, Group 5 was dosed once daily for 5 days and sacrificed 24 h after the last administration. Matched control mice (*n* = 3) to Groups 3 and 5 received tricaprylin only. Liver, lung, small intestine, spleen, colon and kidney tissues were removed, snap frozen in liquid N_2_, and stored at −80 °C until analysis.

### 2.4 DNA adduct analysis by ^32^P-postlabelling

Genomic DNA was isolated from cells or tissue by a standard phenol/chloroform extraction method and stored at −20 °C. DNA adducts were measured in each DNA sample using the butanol enrichment version of the ^32^P-postlabelling method as described previously [32], [35] with minor modifications. Briefly, DNA samples (4 μg) were digested with micrococcal nuclease (120 mU; Sigma, #N3755) and calf spleen phosphodiesterase (40 mU; Calbiochem, #524711), enriched and labelled with 50 μCi [γ-^32^P]ATP (Hartmann Analytic #HP601ND) as reported. 3-NBA-derived DNA adducts were identified as reported [11], [12], [13].

### 2.5 Isolation and culture of primary HUFs

Xpa-WT and Xpa-Null Hupki mouse embryonic fibroblasts (HUFs) were isolated from day 13.5 embryos of intercrosses of *Hupki*^+/+^; *Xpa*^+/−^; *lacZ*^+^ mice according to a standard procedure, as described previously [27]. Isolated HUFs were cultured to 80–90% confluence for 2–3 days in growth medium (Dulbecco’s modified medium (Invitrogen #31966-021) supplemented with 10% fetal bovine serum (Invitrogen #26140-079) and 100 U/mL penicillin and streptomycin (Invitrogen #15140-130) at 37 °C/5% CO_2_/3% O_2_ before preparing frozen stocks (passage 0) and genotyping.

Cells were cultured in growth medium at 37 °C/5% CO_2_ in either 20% or 3% O_2_, adjusted using an incubator fitted with an oxygen sensor and a nitrogen source. Manipulations conducted outside the incubator were performed at 20% O_2_. For passaging cells were detached with 0.05% trypsin-EDTA (Invitrogen #25300-062), suspended in growth media and reseeded at the desired dilution or cell number.

### 2.6 Cell survival assay

Cell survival was assessed using a crystal violet staining assay as described previously [27]. Xpa-WT or Xpa-Null HUFs were seeded into 96-well plates at 2.5–5.0 × 10^3^ cells/well and treated the following day with 3-NBA diluted in growth medium to a highest final concentration of 4 μM (0.1% DMSO). Cells were treated at 37 °C/5% CO_2_/3% O_2_ in 5 replicate wells per concentration of 3-NBA or DMSO (control). Following 24 or 48 h of treatment, cells were rinsed with PBS, stained for 15 min with 0.1% (w/v) crystal violet (Sigma #C3886) in 10% ethanol, washed again with PBS to remove excess dye and then allowed to air-dry. To quantify the crystal violet retained by surviving cells, 100 μL of 50% ethanol was added per well to solubilise the dye and the absorbance at 595 nm was determined using a plate reader. Data are presented as the percentage of A_595nm_ in 3-NBA treated cells relative to that of control cells and are representative of at least three independent experiments.

### 2.7 3-NBA treatment of HUFs for DNA adduct analysis

Xpa-WT and Xpa-Null HUFs were cultured and treated at 37 °C/5% CO_2_ in either 3% or 20% O_2_. HUFs (passage 1, ≤ 7 days in culture) were seeded at 1.0–1.5 × 10^6^ cells/75-cm^2^ flask and treated the following day for 48 h. 3-NBA was diluted in growth medium to a final concentration of 0.1, 0.5, 1.0 or 2.0 μM (0.1% DMSO) and cells were treated in duplicate flasks per condition. Following treatment cells were trypsinised, pelleted and stored at −20 °C until further analysis.

### 2.8 Western blotting

Treated cells were lysed with 62.5 mM Tris–HCl pH 6.8, 500 mM EDTA pH 8.0, 2% sodium dodecyl sulphate (SDS) and 10% glycerol, supplemented with fresh protease inhibitors (78425; Thermo Fisher, UK). The expression of p53, phospho-p53 (S15), p21 and Gapdh in the lysates was assessed by SDS-PAGE and Western blotting as reported previously [36] using the following primary antibodies: anti-p53 (NCL-p53-CM5, Leica Microsystems, UK; 1:1,000), anti-phospho-p53 (#9284, Cell Signaling Technology, UK; 1:1,000); anti-p21 (#556431, BD Bioscience, UK; 1:2,000); and Gapdh (#MAB374, Millipore, UK; 1:25,000).

### 2.9 Hupki mouse embryo fibroblast immortalisation assay (HIMA)

Primary HUFs treated with 3-NBA were immortalised essentially as described previously [27], [37]. Frozen stocks (passage 0) of Xpa-WT and Xpa-Null primary HUFs were thawed and expanded in 175-cm^2^ flasks at 37 °C/5% CO_2_/3% O_2_ for 72 h until 80–90% confluent. Cells were trypsinised, counted and seeded at 2.0 × 10^5^ cells/well into 6-well Corning CellBind^®^ plates. Cells were treated the following day with 1 μM 3-NBA for 48 h (36 cultures, each Xpa genotype) or 2 × 48 h (24 cultures, each Xpa genotype). For cultures receiving 2 treatments, ∼300,000 cells from each culture (Xpa-WT and Xpa-Null) were reseeded following the first 48 h treatment with 3-NBA and then retreated the following day for a further 48 h. Additionally, a set of HUF cultures (54 cultures, each Xpa genotype) were treated with 0.1% DMSO in parallel [27].

Following treatment, as cultures approached confluence, HUFs were subcultured on 6-well Corning CellBind^®^ plates at a dilution of 1:2–1:4. At Day 11 (post-thaw), all cultures were transferred to 20% O_2_ to select for senescence bypass. Cultures were serially passaged at dilutions of 1:2–1:4 until senescence/crisis (ceasing of cell division and enlarged morphology); during crisis cells received fresh media every 3 days. Serial passaging was resumed once regions of dividing cells emerged (immortalised clones). Cultures containing clones were split at dilutions of at least 1:2–1:4 for several passages, followed by further passaging at dilutions up to 1:20. Cultures were expanded to larger flasks (25-, 75-cm^2^) once they appeared homogenous and achieved a doubling rate of ≤ 48 h; frozen stocks were prepared and a portion of cells was pelleted for DNA extraction (≥ passage 12; 8–16 weeks).

### 2.10 *TP53* mutation analysis

DNA for *TP53* mutations analysis was prepared as described recently [27]. Human *TP53*-specific primers (exon 4 to exon 9, including introns, and cycling conditions are described in Supplementary Table 2). PCR amplification products were submitted to Beckman Coulter Genomics (Takeley, UK) for Sanger dideoxy fluorescent sequencing using the sequencing primers indicated in Supplementary Table 2. Sequences were assessed for the presence of *TP53* mutations as described [27]. Variations (*e.g.* single base substitutions, deletions) were assessed using the mutation validation tool available at the IARC *TP53* mutation database (http://www-p53.iarc.fr/MutationValidationCriteria.asp) and could be classified as either homo-/hemi-zygous or heterozygous. Mutations were confirmed by sequencing DNA from an independent sample of cells from the same clone.

### 2.11 *lacZ* mutation assay

Xpa-WT and Xpa-Null primary HUFs (passage 0) carrying the *lacZ* reporter gene were thawed and expanded at 37 °C/5% CO_2_/3% O_2_ for 72 h. Cells (1.0–1.5 × 10^6^) were seeded into 75-cm^2^ flasks (5 flasks per treatment). The following day cells were treated with 3-NBA (0.1, 0.5, 1.0 or 2.0 μM; cytotoxic and sub-cytotoxic concentrations) or solvent control (0.1% DMSO) for 48 h. After treatment, cells from each flask were trypsinised, counted and reseeded at 1.75 × 10^6^ cells into 175-cm^2^ flasks to allow cells to proliferate and fix mutations. After 96 h, when control cultures were ∼90% confluent, cells were trypsinised, pelleted and stored at −20 °C until DNA isolation. DNA was isolated from cells by standard phenol/chloroform extraction. pUR288 (*lacZ*) plasmid rescue and mutant frequency determination were performed as described previously [38], [39], [40]. Briefly, 10–30 μg of DNA were digested with HindIII and then incubated with magnetic beads coated with lacI fusion protein. Plasmids were eluted from the beads using isopropyl-β-D-thiogalactopyranoside (IPTG), circularised at the cohesive HindIII sites using T4 DNA ligase and then electroporated into *E. coli* deficient in β-galactosidase (*lacZ*^−^) and galactose epimerase (*galE*^−^). One thousandth of the transformed bacteria was plated on nonselective, titre plates (containing X-Gal (5- bromo-4-chloro-3-indolyl-β-D-galactopyranoside)) and the remainder was plated onto selective plates (containing the lactose analog P-Gal (phenyl-β-D galactosidase)). Mutant frequencies were calculated as the ratio between the number of mutant colonies on the selective plate to the total number of colonies on the titre plate (times the dilution factor of 1,000).

## 3 Results

### 3.1 DNA adduct formation by 3-NBA in Xpa-WT and Xpa-Null Hupki mice

**Fig. 1:**
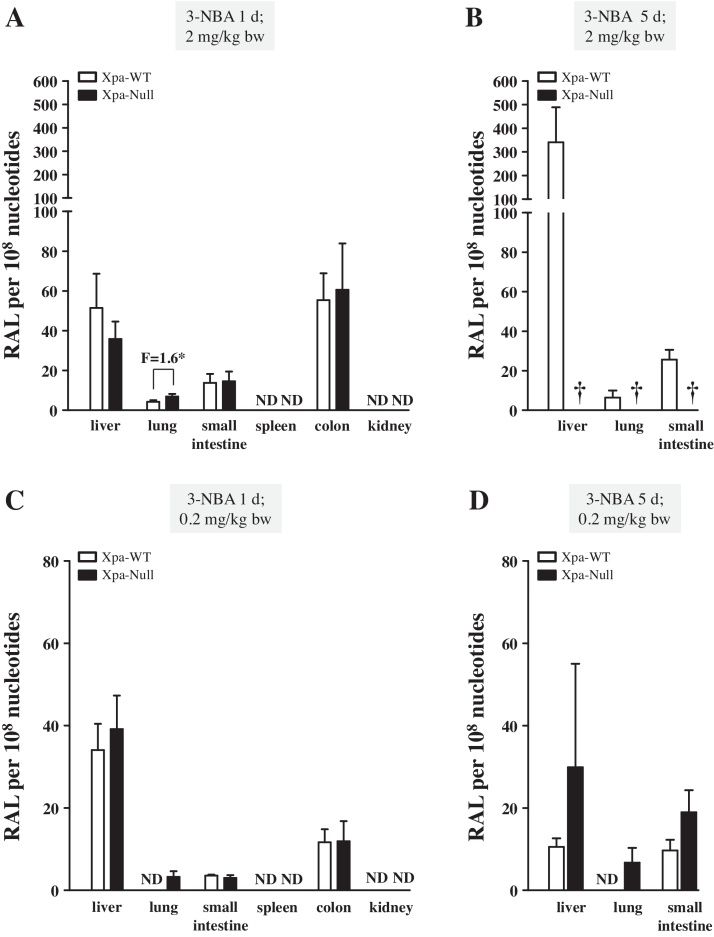
DNA adduct formation in Xpa-WT and Xpa-Null Hupki mice treated with 3-NBA. Mice were treated with 3-NBA (2 or 0.2 mg/kg bw) either once (A) and (C) or once a day for 5 days (B) and (D). DNA adduct levels (RAL, relative adduct labelling) in the indicated tissues were assessed by ^32^P-postlabelling. See Supplementary Fig. 2 for representative autoradiograms showing the adduct profiles in examined tissues. Values represent means ± SD from 3 animals, and each DNA sample was measured by two independent ^32^P-postlabelling analyses. ND = not detected; † **=** not determined due to death of Xpa-Null animals. Statistical analysis, comparing adduct levels in tissues from Xpa-WT and Xpa-Null mice, was performed using the Student’s *t* -test; * *P* < 0.05.

The effect of NER deficiency on 3-NBA-induced DNA adduct formation *in vivo* was assessed by ^32^P-postlabelling (Fig. 1). The DNA adduct pattern induced by 3-NBA (see Supplementary Fig. 2) consisted of up to four adduct spots. These were identified previously [11], [12], [13] as deoxyadenosine (adduct spots 1 and 2) and deoxyguanosine adducts (adduct spots 3 and 4), of which three have been structurally characterised as 2-(2′-deoxyadenosin-*N*^6^ aminobenzanthrone (dA-*N*^6^-3-ABA; spot 1), 2-(2′-deoxyguanosin-*N*^2^-yl)-3-aminobenzanthrone (dG-*N*^2^-3-ABA; spot 3) and *N*-(2′-deoxyguanosin-8-yl)-3-aminobenzanthrone (dG-C8-*N*-3-ABA; spot 4). The structures of these adducts are shown in Supplementary Fig. 1.

One day after a single administration of 2 mg/kg bw 3-NBA, Xpa-Null mice had a 1.6-fold higher level of DNA adducts in the lung compared to Xpa-WT animals but similar DNA adduct levels in the liver, small intestine and colon (Fig. 1A). No adducts were detected in spleen or kidney. The highest levels of adducts were detected in liver (51 ± 17 [Xpa-WT] *versus* 36 ± 9 adducts per 10^8^ nt [Xpa-Null]) and colon (55 ± 13 [Xpa-WT] *versus* 61 ± 23 adducts per 10^8^ nt [Xpa-Null]). While 3-NBA was well tolerated by Xpa-WT mice, all Xpa-Null mice died within 2–3 days of 3-NBA treatment (2 mg/kg bw) (Fig. 1B and Supplementary Fig. 3). 3-NBA-DNA adduct levels persisted in the tissues of Xpa-WT mice 5 days after a single treatment (Supplementary Fig. 3). Further, 3-NBA-DNA adduct levels increased nearly 6-fold in the liver and ∼2-fold in the small intestine of mice that were treated daily for 5 consecutive days (Fig. 1B).

As Xpa-Null mice were highly sensitive to treatment with 3-NBA at 2 mg/kg bw, animals were subsequently treated with a 10-fold lower dose (0.2 mg/kg bw) in a follow-up experiment (Fig. 1C and D). In mice treated once with 0.2 mg/kg bw 3-NBA, 3-NBA-DNA adducts were detectable in the lungs of Xpa-Null mice but not Xpa-WT mice; no significant difference between DNA adduct formation in Xpa-WT and Xpa-Null animals was found in other organs with detectable levels of 3-NBA-DNA adducts (*i.e.* liver, small intestine, and colon). Following a single treatment with 0.2 mg/kg bw 3-NBA, DNA adduct levels in the lung, small intestine and colon were at least 2-fold lower, or undetectable, (Fig. 1C) compared with tissues from mice that received 2 mg/kg bw (Fig. 1A). In contrast, adduct levels in the liver were similar after low or high dose treatment. Both Xpa-WT and Xpa-Null mice survived 5 daily treatments of 0.2 mg/kg bw 3-NBA. Again 3-NBA-DNA adducts were detectable in the lungs of Xpa-Null mice but not in Xpa-WT mice, indicating that 3-NBA-DNA adducts were repaired by NER. In the other two tissues (liver and small intestine) assessed from Group 5 animals, a trend for higher DNA adduct levels in Xpa-Null mice than in Xpa-WT mice was found (Fig. 1D), but the observed difference did not reach statistical significance. Interestingly, adduct levels in the livers of both genotypes from Group 5 mice were lower than those detected for Group 4 animals (Fig. 1C).

### 3.2 3-NBA-induced DNA adduct formation in Xpa-WT HUFs cultured in 3% or 20% O_2_

**Fig. 2:**
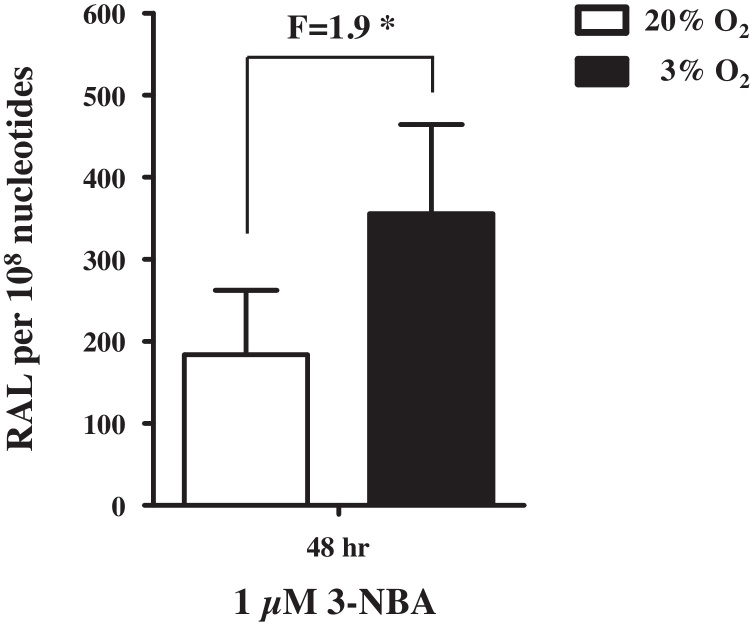
3-NBA-induced DNA adduct formation in HUFs cultured at 20% or 3% O_2_. Xpa-WT HUFs cultured in 20% or 3% O_2_ were treated for 48 h with 1 μM 3-NBA. DNA adduct formation (RAL, relative adduct labelling) was assessed by ^32^P-postlabelling. Values represent means ± SD of two biological replicates where each DNA sample was measured by two independent ^32^P-postlabelling analyses. Statistical analysis, comparing DNA adduct formation at 20% and 3% O_2_, was performed using the Student’s *t* -test; * *P* < 0.05.

We then went on to examine DNA adduct formation in HUFs treated with 3-NBA. Previously, we showed that when HUFs were cultured in physiological O_2_ (3%) their replicative potential was enhanced [27]. This suggested that cells could be cultured in 3% O_2_ before and during mutagen treatment to increase the number of cells available for experiments. Cells would then be transferred to 20% O_2_ to select for mutated cells capable of senescence bypass. The impact of oxygen on 3-NBA metabolism and 3-NBA-induced DNA damage is unknown. Thus, we treated primary Xpa-WT HUFs grown in 3% or 20% O_2_ with 1 μM 3-NBA for 48 h to assess DNA adduct formation. We found that DNA adduct levels were 2-fold higher in HUFs treated with 3-NBA in 3% O_2_ compared with cells treated in 20% O_2_ (Fig. 2). Therefore, all subsequent 3-NBA treatments of HUFs in this study were performed in 3% O_2_.

### 3.3 DNA adduct formation and survival of 3-NBA-treated Xpa-WT and Xpa-Null HUFs

**Fig. 3:**
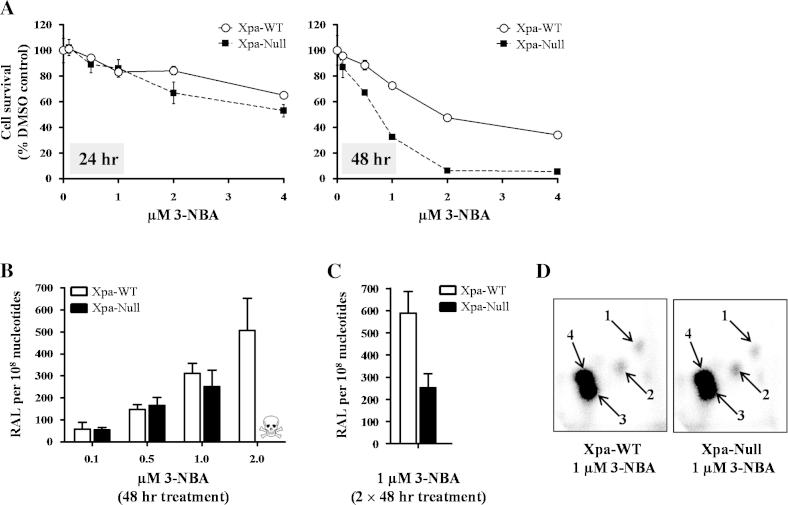
Survival and DNA adduct formation in Xpa-WT and Xpa-Null HUFs treated with 3-NBA. (A) Cells were treated for 24 or 48 h with the indicated doses of 3-NBA and survival was measured using a crystal violet staining assay. Cells treated with 0.1% DMSO only served as control. Mean values are presented as% of control ± SD of 5 replicate wells and are representative of at least three independent experiments (variation ≤ 15%). (B) and (C) Cells were treated with 3-NBA (as indicated) for 48 h or 2 × 48 h and DNA adduct formation (RAL, relative adduct labelling) was assessed by ^32^P-postlabelling. Values represent means ± SD of two biological replicates where each DNA sample was measured by two independent ^32^P-postlabelling analyses. Due to severe loss of viability, DNA adduct formation was not assessed in Xpa-Null cells treated with 2 μM 3-NBA (indicated by skull and crossbones). (D) Autoradiographic profiles of DNA adducts obtained in 3-NBA-treated HUFs; arrows indicate spot 1: 2-(2′-deoxyadenosin-*N*^6^-yl)-3-aminobenzanthrone (dA-*N*^6^-3-ABA), spot 3: 2-(2′-deoxyguanosin-*N*^2^-yl)-3-aminobenzanthrone (dG-*N*^2^-3-ABA) and spot 4: *N*-(2′-deoxyguanosin-8-yl)-3-aminobenzanthrone (dG-C8-*N*-3-ABA). Spot 2 is a deoxyadenosine adduct that has not yet been structurally characterised. The origins, in the bottom left-hand corners, were cut off before exposure.

Xpa-WT and Xpa-Null HUFs were compared for their sensitivity (*i.e.* cell survival) to 3-NBA after 24 or 48 h treatment (Fig. 3A). Xpa-Null HUFs were more sensitive to 3-NBA treatment; however, the decrease in cell survival was more pronounced after 48 h. After 24-h treatment with 1 μM 3-NBA, 80% of Xpa-Null cells had survived (similar for Xpa-WT), whereas after 48-h treatment, the surviving fraction was ∼30% of control (∼70% for Xpa-WT). At higher doses of 3-NBA (2–4 μM), most Xpa-Null cells were dead after 48 h, whereas at least 40% of Xpa-WT cells had survived.

Next, DNA adduct formation induced by 3-NBA was assessed in Xpa-WT and Xpa-Null HUFs. Cells were treated with 0.1–2.0 μM of 3-NBA for 48 h (Fig. 3B). Although proficient in NER, Xpa-WT HUFs accumulated similar levels of DNA adducts (up to ∼300 adducts per 10^8^ nt) to Xpa-Null HUFs after treatment with 0.1–1.0 μM 3-NBA. However, while Xpa-WT HUFs accrued 507 ± 144 adducts per 10^8^ nt after 2 μM 3-NBA treatment, Xpa-Null HUFs did not survive treatment at that concentration. We concluded from these data that a 48-h treatment with 1 μM 3-NBA induced substantial DNA damage in both Xpa-WT and Xpa-Null HUFs (Xpa-WT: ∼300 adducts per 10^8^ nt; Xpa-Null: ∼250 adducts per 10^8^ nt) while preserving a population of viable cells that could proliferate and accumulate mutations (Xpa-WT: ∼85% survival; Xpa-Null: ∼30% survival). Further, as previous HIMAs have often employed repeated treatments with a mutagen [18], [41], we also assessed DNA adduct formation in HUFs following 2 × 48-h treatments with 1 μM 3-NBA (Fig. 3C). In Xpa-WT HUFs, two treatments with 1 μM 3-NBA increased the DNA adduct level ∼2-fold over the level induced by a single treatment, whereas in Xpa-Null HUFs DNA adduct levels were nearly the same after one or two treatments. Cell survival of both Xpa-WT and Xpa-Null HUFs was ∼2-fold lower following repeated treatment with 3-NBA than after a single treatment (data not shown).

The adduct patterns induced by 3-NBA (Fig. 3D) consisted of four adduct spots that were identical to those observed in several organs of Xpa-WT and Xpa-Null Hupki mice treated with 3-NBA *in vivo* (see Supplementary Fig. 2). No DNA adducts were detected in HUFs treated with solvent (DMSO) only (data not shown).

### 3.4 Induction of the p53 pathway in Xpa-WT and Xpa-Null HUFs after 3-NBA treatment

Induction of p53 in 3-NBA-treated Xpa-WT and Xpa-Null HUFs was assessed by immunoblotting (Supplementary Fig. 4). Expression of p53, and phosphorylation on serine 15, was highly induced after treatment of Xpa-Null HUFs with 0.5 and 1.0 μM 3-NBA, coupled with a moderate induction of p21, a p53 transcriptional target. Similar treatment of Xpa-WT cells only slightly induced expression and phosphorylation of p53, and resulted in a very modest increase in p21 expression. The highest dose of 3-NBA (2 μM) tested in Xpa-WT HUFs strongly induced phosphorylation of p53 and increased p53 expression to a level similar to that observed in Xpa-Null HUFs treated with 0.5 μM 3-NBA. HUFs were also treated with 0.25 μM BaP for comparison. BaP treatment strongly induced p53 expression and phosphorylation in Xpa-Null cells, whereas the p53 response was much weaker in the Xpa-WT cells.

### 3.5 *TP53* mutations induced by 3-NBA in Xpa-WT and Xpa-Null HUFs

#### 3.5.1 Mutation frequency

We next examined the effect of NER-deficiency on 3-NBA-induced *TP5*3 mutagenesis using the HIMA. Xpa-WT and Xpa-Null HUFs were treated either 1 × 48 h or 2 × 48 h with 1 μM 3-NBA and then serially passaged for ≥12 passages (8–16 weeks) until established into immortalised clones. Untreated cells were immortalised in parallel [27]. Mutations in exons 4–9 and adjacent introns of the human *TP53* sequence in immortal HUF clones are shown in Table 1, Table 2.

As described previously [27], *TP53* mutations were only detected in two spontaneously immortalised HUF clones from untreated cultures of either genotype (3.7%) (Table 1). Following 3-NBA treatment the frequency of *TP53*-mutated clones increased to 18.3% in both Xpa-WT and Xpa- Null cultures. A similar frequency of *TP53*-mutated clones was generated following a single or double treatment with 3-NBA. A total of 14 *TP53* mutations were detected in 11 immortal clones derived from 3-NBA-treated Xpa-WT cultures (three clones contained two mutations each) and 12 *TP53* mutations were detected in 11 immortal clones derived from 3-NBA-treated Xpa-Null cultures (1 clone harboured 2 mutations). Treatment with 3-NBA clearly increased the frequency of *TP53* mutations over that of untreated cultures, but, interestingly, *Xpa*-deficiency did not further increase the mutation frequency induced by 3-NBA.

#### 3.5.2 Mutation pattern and sequence context

**Fig. 4:**
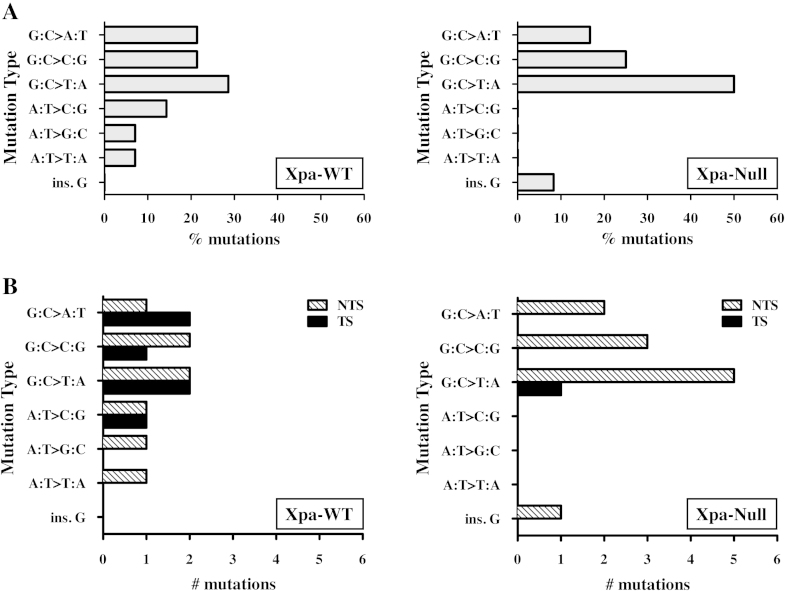
*TP53* mutation pattern and strand bias in 3-NBA-treated Xpa-WT and Xpa-Null HUFs. (A) The % of each type of single base substitution or insertion is shown. (B) The number of each type of single base substitution or insertion occurring on the non-transcribed strand (NTS) or transcribed strand (TS) is shown.

All but one of the *TP53* mutations (an insertion) were single base substitutions and occurred at splice sites or in coding sequences, resulting in an amino acid substitution. No silent or nonsense mutations were found. The predominant mutation type identified in clones from 3-NBA-treated cultures was G:C > T:A transversion, representing 29% of the mutations in Xpa-WT clones and 50% of the mutations in Xpa-Null clones (Table 1 and Fig. 4). With the exception of one clone that contained an insertion (XN-3N-151), all of the remaining mutations found in the 3-NBA-treated Xpa-Null clones also occurred at G:C base pairs; 25% were G:C > C:G transversions and 17% were G:C > A:T transitions. G:C > C:G and G:C > A:T base changes were similarly found in 3-NBA-treated Xpa-WT clones (∼20% for both mutation types). Guanines at CpG dinucleotides were often mutational targets in both Xpa-WT and Xpa-Null clones. Overall, 40% of G:C > A:T transitions and 60% of G:C > T:A transversions occurred at CpG sites (Supplementary Table 3). G:C > G:C transversions, on the other hand, occurred most frequently in a sequence context of 5′G-G-C3′.

In contrast to 3-NBA-treated Xpa-Null clones, ∼30% of the mutations in Xpa-WT clones occurred at A:T base pairs. This mutation type was not found in any 3-NBA-treated Xpa-Null clones. In untreated control cultures, three of the four *TP53* mutations identified were A:T > C:G base substitutions. This mutation type was not found in 3-NBA-treated Xpa-Null cultures, but was found in 2 clones from 3-NBA-treated Xpa-WT HUFs (XW-3N-15 and -54; Table 2); notably, these clones also harboured a second *TP53* mutation.

#### 3.5.3 Mutational strand bias

As the non-transcribed strand of *TP53* is repaired more slowly than the transcribed strand in cells with normal NER capacity, mutations induced by carcinogens such as BaP or UV are preferentially found on the non-transcribed strand [42], [43]. In cells deficient in TC-NER, however, an increase in mutations on the transcribed strand may occur [44]. Indeed, we previously showed that BPDE-induced *TP53* mutations were more frequently induced on the transcribed strand in Xpa-Null HUFs compared with Xpa-WT HUFs [27]. Interestingly, here we did not find an excess of 3-NBA-induced *TP53* mutations on the transcribed strand in Xpa-Null HUFs (Table 2, Fig. 4). In fact, only one Xpa-Null clone harboured a mutated guanine on the transcribed strand (G:C > T:A at codon 273) compared with five such mutations in Xpa-WT clones (G:C > T:A at codons 178 and 179; G:C > C:G at codon 161; G:C > A:T at codons 248 and 282).

#### 3.5.4 Codon distribution and effect of mutations on p53 function

3-NBA-induced *TP53* mutations occurred in all sequenced exons (4–9). Only one codon, 249, was mutated in both Xpa-WT and Xpa-Null HUFs. In both cases the mutation type at codon 249 was G:C > C:G, although a different guanine was targeted in the Xpa-WT and Xpa-Null clones (AGG to ACG *vs* . AGG to AGC, respectively). Of the seven residues in the p53 core DNA binding domain that make direct contact with DNA bases (K120, S241, R248, R273, A276, C277, R280), two were mutated in Xpa-Null clones (R273 and C277) and two were mutated in Xpa-WT clones (K120 and R248) [45]. Additionally, several mutations were induced at residues that support the structure of the DNA binding surface of p53: G245 and R249 in Xpa-Null clones; R175, R249 and R282 in Xpa-WT clones. H179, a residue important for coordinating zinc-binding, was mutated in two Xpa-WT clones.

The majority of the 3-NBA-induced *TP53* mutations were classified as non-functional, based on a comprehensive study of mutant transactivation function in yeast [46]; five mutations were classified as partially functional. Three of the mutations with partial functionality (A161P, A161G, H179P) were detected in clones harbouring two mutations (XW-3N-14, -15, 54), where the second mutation was non-functional (R249S, L194R, and H178Q, respectively). This suggests, as discussed previously [27], that the HIMA selects for clones harbouring non-functional p53 mutants and that p53 mutants retaining partial functionality may not be capable of senescence bypass.

#### 3.5.5 Comparison of 3-NBA-induced TP53 mutations with mutations found in human cancer

**Fig. 5:**
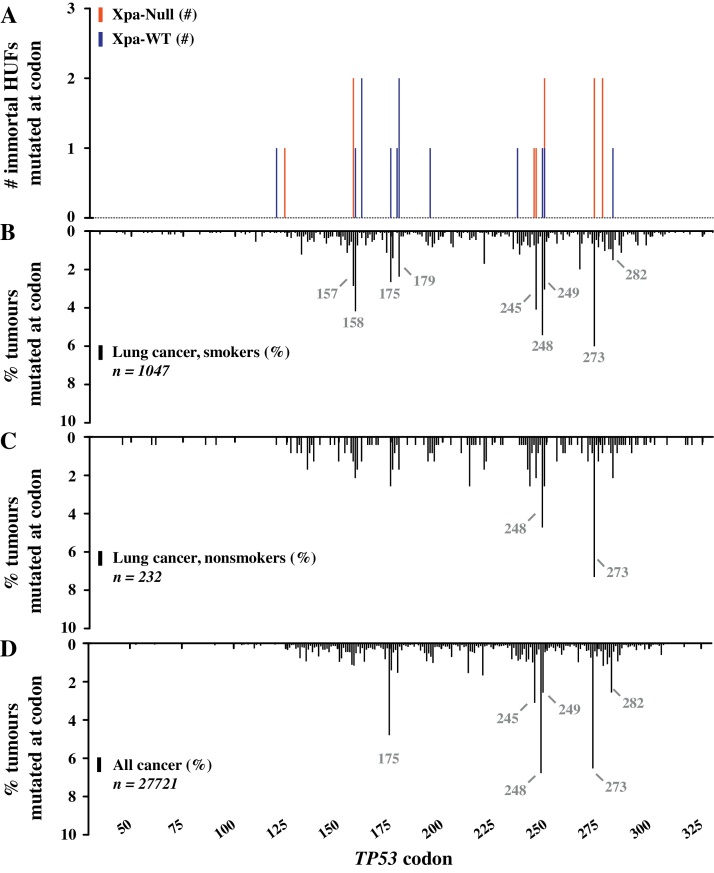
The spectrum of *TP53* mutations induced by 3-NBA in Xpa-WT and Xpa-Null HUFs compared with mutations found in human tumours. Shown are (A) the number of mutations at each codon (within exons 4–9) in the *TP53* gene induced by 3-NBA in HUFs compared with the frequency of mutation at each codon in (B) lung cancer of smokers (exclusions: radon, asbestos, mustard gas, and coal [65], [66], (C) lung cancer of non- and passive-smokers (exclusions: radon, asbestos, mustard gas, and coal [65], [66] and (D) cancer overall. Reference for the codon distribution of *TP53* mutations in human cancer: IARC *TP53* Mutation Database, R17 (November 2013). Mutation hotspots are indicated in grey.

The *TP53* mutations induced by 3-NBA were compared with *TP53* mutations found in human cancer, as catalogued in the IARC *TP53* mutation database, version R17 (Fig. 5). The frequency of each mutation was assessed in lung cancer of smokers and non-smokers, as well as cancer overall (Fig. 5B–D). Each mutation induced by 3-NBA in this study has been found in at least one human tumour. Further, all codons and splice sites mutated by 3-NBA have been targeted in lung tumours, including 16/25 identical mutations.

Interestingly, all of the most frequently mutated hotspot codons in smokers’ lung cancer (157, 158, 175, 179, 245, 248, 249, 273, 282) or cancer overall (175, 245, 248, 249, 273, 282) were mutated following 3-NBA treatment in the HIMA (Fig. 5A). Six hotspots were mutated in Xpa-WT clones (158, 175, 179, 248, 249, 282) and four hotspots were mutated in Xpa-Null clones (157, 245, 249, 273). In fact, the mutation spectrum induced by 3-NBA in the current HIMA closely mirrors that of smokers' lung cancer (Fig. 5B).

Specific mutations induced by 3-NBA in this HIMA that are most frequently detected in human cancer include GTC to TTC at codon 157, CGC to CTC at codon 158, and CGG to TGG at codons 248 and 282 (Supplementary Table 4). Interestingly, those four hotspot codons have never been reported as mutated in spontaneously immortalised HUFs, suggesting that they may represent specific targets for carcinogen-induced mutations. Further, G:C > A:T transversions located at codons 157, 158, 175, 273 and 277, all of which were induced by 3-NBA in this study, are more commonly found in lung cancer than in cancer overall. Therefore, one could speculate that these mutations in lung tumours may indicate exposure to compounds such as nitro-PAHs or PAHs found in air pollution or tobacco smoke.

#### 3.5.6 Comparison to mutations from previous HIMAs

The *TP53* mutations induced by 3-NBA in the current study were compared to the mutations generated in previous HIMAs (Supplementary Table 5). Four codons (236, 244, 245, 273) mutated by 3-NBA in the current assay were also targeted by 3-NBA in a previous assay [18]. Identical mutations at three codons occurred in both 3-NBA studies (236: TAC to TGC; 244: GGC to GCC; 273: CGT to CTT). Further, identical mutations to those induced by 3-NBA at five codons in the current study were also induced by treatment with BaP/BPDE (157: GTC to TTC; 158: CGC to CTC; 245: GGC to CGC; 249: AGG to AGC; 273: CGT to AGT or CTT). While aristolochic acid I (AAI) and UV also targeted several similar hotspot codons to 3-NBA (AAI: 158, 179, 245, 249, 273; UV: 179, 248, 249) the mutations induced at these sites by AAI and UV were not identical to those generated by 3-NBA.

### 3.6 3-NBA-induced mutation of *lacZ* reporter gene in Xpa-WT and Xpa-Null HUFs

The impact of NER deficiency on 3-NBA-induced mutagenesis in HUFs was further assessed at the *lacZ* reporter gene. The Xpa-WT and Xpa-Null HUFs utilised in this study harbour a pUR288 plasmid locus containing *lacZ*. Within a few days of treating HUFs with a carcinogen, the pUR288 plasmid can be recovered from HUF DNA and amplified in *E. coli* to determine *lacZ* mutant frequencies.

**Fig. 6:**
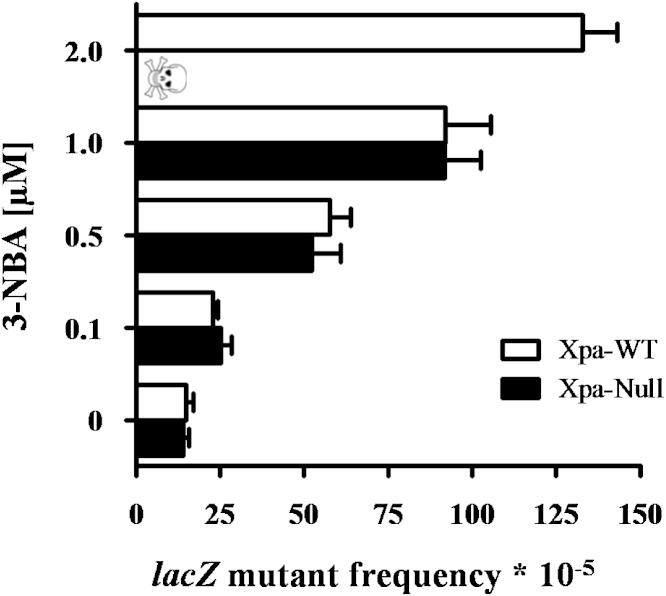
Mutant frequency at the *lacZ* locus in Xpa-WT and Xpa-Null HUFs treated with 3-NBA. HUFs were treated for 48 h with the indicated concentrations of 3-NBA (or 0.1% DMSO solvent control) and then allowed to proliferate for 4 days to fix DNA damage into mutations. *LacZ* mutant frequencies were calculated as the number of mutant colonies per number of recovered transformants.

Xpa-WT and Xpa-Null HUFs were treated with 0.1–2.0 μM 3-NBA for 48 h, and then assessed for frequency of *lacZ* mutations after 4 days of mutation fixation (Fig. 6). A background mutant frequency of 14–15 × 10^−5^ was detected in both Xpa-WT and Xpa-Null HUFs. Following treatment with increasing concentrations of 3-NBA, a dose-dependent increase in *lacZ* mutant frequency was observed. Interestingly, as found for *TP53* in the HIMA, the mutant frequency of *lacZ* was very similar between Xpa-WT and Xpa-Null HUFs at all concentrations, with the exception of cells treated with 2 μM 3-NBA, where the lack of viable cells precluded the assessment of mutants in Xpa-Null HUFs. Treatment with 1 μM 3-NBA increased the mutant frequency ∼6-fold over the background to 92 × 10^−5^ in Xpa-WT and Xpa-Null HUFs. The mutant frequency in Xpa-WT HUFs rose to ∼9-fold over background (133 × 10^−5^) following treatment with 2 μM 3-NBA.

## 4 Discussion

In this study, *TP53* and *lacZ* mutagenesis by the carcinogenic air pollutant 3-NBA was assessed in Xpa-WT and Xpa-Null HUFs. As Xpa is required for the removal of bulky adducts from the DNA by NER, it was hypothesised that greater persistence of 3-NBA-DNA adducts in Xpa-Null HUFs would lead to increased mutation frequency. However, a similar frequency of 3-NBA-induced *TP53* and *lacZ* mutations was detected in Xpa-WT and Xpa-Null HUFs.

Xpa-Null HUFs and Hupki mice were much more sensitive to 3-NBA than their Xpa-WT counterparts. A pronounced decrease in survival was observed for Xpa-Null HUFs treated with the highest concentrations of 3-NBA and Xpa-Null Hupki mice died within 2–3 days of receiving 2 mg/kg bw 3-NBA. A similar response was found previously for Xpa-Null HUFs and Hupki mice treated with BaP [27]. Further, this sensitivity to genotoxicants was also shown for Xpa-Null mice with wild-type *Trp53* after exposure to UV or 2-amino-1-methyl-6-phenylimidazo[4,5-*b*]pyridine (PhIP) [33], [47]. While Xpa-Null and Csb (Cockayne syndrome B)-Null cells, both deficient in TC-NER, undergo apoptosis after DNA damage induced by UV or BaP, Xpc-Null cells or mice, deficient only in GG-NER, do not [48], [49]. The inability of TC-NER-deficient cells to remove DNA lesions from the transcribed strand of active genes is a strong trigger for the induction of apoptosis [50]. Lesions that persist on the transcribed strand can block the progression of RNAPII transcription complexes, which may subsequently collide with DNA replication forks during S-phase [51]. Therefore, the sensitivity of Xpa-Null HUFs and Hupki mice to 3-NBA could be due to the blocking of RNAPII progression by 3-NBA-DNA adducts. In this context it is noteworthy that a recent study found that 3-NBA-DNA adducts markedly block DNA replication [52]. The replication frequency of dG-C8-*N*-3-ABA was ∼17% compared with ∼33% for dG-*N*^2^-3-ABA and ∼43% for dA-*N*^6^-3-ABA [52].

Despite increased sensitivity to 3-NBA, Xpa-WT and Xpa-Null HUFs accumulated similar 3-NBA-DNA adduct levels. Further, 24 h after a single administration of 3-NBA, a difference in 3-NBA-DNA adduct formation between Xpa-WT and Xpa-Null Hupki mice was only observed in the lung (see Fig. 1). Previously, DNA adduct levels induced by BaP were found to be higher in several tissues (spleen, colon, kidney) in Xpa-Null relative to Xpa-WT Hupki mice [27]. These results indicate that repair of bulky DNA adducts may be tissue- and lesion-specific.

The fact that Xpa-Null HUFs did not exhibit an increase in 3-NBA-induced mutation frequency at the *TP53* and *lacZ* loci nor an increase in DNA adduct levels, compared to Xpa-WT HUFs, suggests that, contrary to expectation, 3-NBA-derived DNA adducts may not be recognised and repaired by GG-NER. GG-NER is initiated following damage recognition by XPC/hHR23B and may occur with varying efficiency, depending on the structure and sequence context of the lesion [53]. Recognition of bulky adducts by XPC/hHR23B is correlated with the degree of thermodynamic destabilisation induced by the lesion [54], [55]. Lesions that distort and destabilise the DNA helix locally (*e.g.* pyrimidine (6–4) pyrimidone photoproducts [6-4PPs] or 10-(deoxyguanosin-*N*^2^-yl)-7,8,9-trihydroxy-7,8,9,10-tetrahydrobenzo[*a*]pyrene [dG-*N*^2^-BPDE]) are repaired efficiently by GG-NER [56], [57], whereas lesions that distort DNA to a lesser degree (*e.g.* cyclobutane pyrimidine dimers [CPDs]) or that stabilise DNA (*e.g.* 3-(deoxyguanosin-*N*^2^-yl)-2-acetylaminofluorene [dG-*N*^2^-AAF] or 8,9-dihydro-8-(2,6-diamino-4-oxo-3,4-dihydropyrimid5-yl-formamido)-9-hydroxyaflatoxin B_1_ [AFB_1_-FAPY]) are repaired less efficiently [58], [59].

It was recently shown that the major DNA adduct formed after metabolic activation of 3-NBA, dG-*N*^2^-3-ABA, had a stabilising effect when incorporated into a synthetic oligonucleotide [60]. The dG-*N*^2^-3-ABA adduct-containing duplex formed a regular double helical structure, with the adduct residing in the minor groove. Therefore, it is possible that the thermodynamic stability of dG- *N*^2^-3-ABA, which is the most abundant and persistent 3-NBA-DNA adduct formed *in vivo* [61], may indeed prevent its recognition and removal by GG-NER. It is important to consider, however, that metabolic activation of 3-NBA generates 4 major adducts, including dG-*N*^2^-3-ABA (see Supplementary Fig. 1) with ∼80% of adducts occurring at deoxyguanosine and ∼20% at deoxyadenosine [9], [11]; the thermodynamic stability of the other moieties is unknown. A recent study evaluating the formation and removal of dA-*N*^6^-3-ABA, dG-*N*^2^-3-ABA and dG-C8-*N*-3-ABA in human cells found that dA-*N*^6^-3-ABA and dG-C8-*N*-3-ABA were repaired more efficiently than dG-*N*^2^-3-ABA [52]. After a 24-hour period ∼85% of the dG-*N*^2^-3-ABA adducts remained, whereas only ∼50% of the dG-C8-*N*-3-ABA adducts and ∼40% of dA-*N*^6^-3-ABA adducts were present in the cells [52].

In contrast to our findings, however, are the results of an earlier study where a *supF* shuttle vector plasmid was treated with *N*-Aco-3-ABA, a reactive metabolite of 3-NBA, and then propagated in NER-proficient and -deficient human fibroblasts (WI-38-VA13 and XP2OS(SV), respectively) [16]. Nearly 2-fold more *supF* mutations were induced in plasmids recovered from the XP2OS(SV) cells compared with the WI-38-VA13 cells. Using a polymerase stop assay, the authors determined that 92% of the *N*-Aco-3-ABA-induced DNA adducts were located at G:C base pairs in *supF*. DNA adducts were not measured in this study, however, therefore their exact composition, such as the proportion of dG-*N*^2^-3-ABA adducts, is unknown. Interestingly, a higher frequency of *supF* mutations induced in the XP2OS(SV) cells occurred at A:T base pairs, compared with the NER-proficient cell line (34% *versus* 17%, respectively) [16]. The authors concluded that dA-3-ABA adducts may be more readily recognised by NER than dG-3-ABA adducts, leading to an increase in mutations at A:T sites in NER-deficient cells. However, this was not the case for *TP53* mutations induced by 3-NBA in Xpa-Null HUFs. While ∼30% of the 3-NBA-induced *TP53* mutations in Xpa-WT HUFs occurred at A:T base pairs, all of the mutations in Xpa-Null HUFs occurred at G:C base pairs.

There are several possible explanations for the differences between our findings and those of Nishida et al. [16]. Firstly, the *supF* gene is not transcribed in human cells, therefore dA-3-ABA adducts in *supF* would not cause RNAPII blockage or apoptosis in XP2OS(SV) cells, but could instead induce mutations. Further, the relative proportion of individual 3-NBA-derived DNA adducts in our systems may be different. The adducts detected in the present study were generated following enzymatic activation of 3-NBA in HUFs, whereas the *supF* shuttle vector was directly reacted with *N*-Aco-3-ABA *in situ* prior to cell transfection. As discussed above, DNA repair efficiency of the four major 3-NBA-derived DNA adducts varies [52], ultimately influencing mutagenesis. Additionally, it could be that 3-NBA-derived DNA adducts are recognised and repaired differently in human cells and mouse cells, or that GG-NER of adducts contained on plasmids operates differently to GG-NER of adducted genomic DNA.

Most 3-NBA-induced mutations occurred at G:C base pairs, with G:C > T:A transversions the predominant mutation type in both Xpa-WT and Xpa-Null HUFs. This is in agreement with the results of an earlier HIMA using 3-NBA performed by vom Brocke et al. [18]. Of note, the dG-*N*^2^-3-ABA and dG-C8-*N*-3-ABA adducts are the predominant 3-NBA-DNA adducts formed in HUFs and both lesions have recently been shown to induce G:C > T:A transversions in site-specific mutagenesis assays in human cells [52]. Four codons were targeted by 3-NBA in both our study and that of vom Brocke et al. (236, 244, 245, 273) [18]. We found that 32% (8/25) of unique *TP53* mutations occurred at CpG sites, compared with only 14% (3/22) in the previous HIMA using 3-NBA. Interestingly, we also found that all of the CpG sites targeted by 3-NBA are hotspots for mutation in human cancer, including smokers' lung cancer (*i.e.* codons 157, 158, 175, 245, 248, 273, 282). Carcinogens such as BPDE show enhanced adduct formation at many of these sites (codons 157, 158, 245, 248, 273) [62], [63]. Our results suggest that metabolites of 3-NBA may also exhibit preferential binding at such sites, although this would have to be confirmed in future studies. The existence of mutation hotspots in cancer can also be influenced by other factors besides enhanced carcinogen binding. These sites may be more resistant to repair or may be more readily bypassed by error-prone translesion polymerases. Additionally, it is possible that these mutations are highly selected for due to their deleterious impact on the function of p53 or their gain-of-function properties [20]. Nevertheless, the spectrum of mutations generated in this study indicates that similar factors influence *TP53* mutagenesis in the HIMA and human cancer.

For the first time, we were able to assess mutagenesis at a *lacZ* reporter gene in HUFs in addition to *TP53* mutagenesis. This allowed us to compare the frequency of 3-NBA-induced *TP53* mutations, detected following a several-month selection process in mammalian cells, to the frequency of *lacZ* mutations generated, but not subjected to selection, in the same cells. In future studies having *lacZ* integrated into HUFs could aid in the optimisation of carcinogen treatment conditions, where the mutagenic activity of an agent could be determined at the *lacZ* locus prior to initiating the more laborious HIMA. The data generated in the present study, comparing 3-NBA-induced mutagenesis of *TP53* and *lacZ* in HUFs, may serve as an initial point of reference for identifying a level of *lacZ* mutation frequency in primary HUFs commensurate with *TP53* mutation frequency in immortalised clones.

While a lack of recognition of 3-NBA-DNA adducts by GG-NER could explain why *TP53* mutant frequency was not increased in Xpa-Null HUFs, as discussed above, it is unclear why these cells did not at least harbour more mutations on the transcribed strand compared with Xpa-WT HUFs, as was observed previously for BPDE [27]. It will be important to examine additional mutagens in future studies, particularly those with firm evidence for removal by NER (*e.g.* UV). One drawback of using Xpa-Null HUFs in the HIMA is their extreme sensitivity to genotoxicants that block RNAPII. This sensitivity necessitates treating Xpa-Null HUFs with a much lower concentration of carcinogen than Xpa-WT cells can tolerate, and therefore lowers the level of premutagenic DNA damage. Xpa-Null HUFs may be particularly useful for improving HIMA mutation frequencies when assessing mutagens that do not generate high levels of DNA damage in HUFs, if such damage is recognised by GG-NER. Furthermore, Xpa-Null HUFs may be useful in determining the role NER plays in shaping *TP53* mutagenesis, rather than simply increasing mutation frequencies.

Although NER-deficient HUFs did not increase the frequency of 3-NBA-induced mutations in the HIMA, data from this study contribute to our understanding of the *TP53* mutation spectrum induced by 3-NBA and further indicate that 3-NBA-derived DNA adducts may evade removal by GG-NER. The persistence of 3-NBA adducts in DNA may be an important factor in its mutagenicity [9], [52], [61]. 3-NBA is an air pollutant, predominantly originating from diesel emissions [2], [6]. Occupational diesel exhaust exposure has been shown to increase the risk of developing lung cancer [2], [64], and compounds such as 3-NBA may contribute to the process. The current evidence from experimental models indicates that 3-NBA is a potent mutagen and carcinogen, but it will be important to assess *TP53* mutations in tumour samples from diesel exhaust-exposed individuals in order to assess more fully what impact this compound has on human carcinogenesis.

## Figures and Tables

**Table 1:** Summary of *TP53* mutation frequency and pattern detected in immortalised clones of Xpa-WT and Xpa-Null HUFs treated with 3-NBA.

3-NBA	Xpa-WT	Xpa-Null	control	Xpa-WT	Xpa-Null
Total 3-NBA-treated HUF cultures (#)	60	60	Total 0.1% DMSO-treated HUF cultures (#)	54	54
*TP53*-mutant immortalised clones (#)	11	11	*TP53*-mutant immortalised clones (#)	2	2
Total *TP53* mutations detected (#)	14	12	Total *TP53* mutations detected (#)	2	2
Frequency of *TP53*-mutant clones	18.3% (11/60)	18.3% (11/60)	Frequency of *TP53*-mutant clones	3.7% (2/54)	3.7% (2/54)
Mutations on the transcribed strand	42.8% (6/14)	8.3% (1/12)	Mutations on the transcribed strand	50% (1/2)	100% (2/2)
**TP*53* mutation pattern			*TP53* mutation pattern		
G to A	21.4% (3/14)	16.7% (2/12)	G to A	0% (0/2)	0% (0/2)
G to C	21.4% (3/14)	25% (3/12)	G to C	50% (1/2)	0% (0/2)
G to T	28.6% (4/14)	50% (6/12)	G to T	0% (0/2)	0% (0/2)
A to C	14.3% (2/14)	0% (0/12)	A to C	50% (1/2)	100% (2/2)
A to G	7.1% (1/14)	0% (0/12)	A to G	0% (0/2)	0% (0/2)
A to T	7.1% (1/14)	0% (0/12)	A to T	0% (0/2)	0% (0/2)
Ins. G	0% (0/14)	8.3% (1/12)	Ins. G	0% (0/2)	0% (0/2)
*TP53* mutation pattern at CpG sites			*TP53* mutation pattern at CpG sites		
Total G mutations at CpG	40% (4/10)	41.7% (5/12)	Total G mutations at CpG	0% (0/1)	0% (0/0)
G to A at CpG	66.7% (2/3)	0% (0/2)	G to A at CpG	0% (0/0)	0% (0/0)
G to C at CpG	0% (0/3)	33.3% (1/3)	G to C at CpG	0% (0/1)	0% (0/0)
G to T at CpG	50% (2/4)	66.7% (4/6)	G to T at CpG	0% (0/0)	0% (0/0)
Ins. G at CpG	0% (0/0)	0% (0/1)	Ins. G at CpG	0% (0/0)	0% (0/0)

**Table 2:** *TP53* mutations induced by 3-NBA in immortalised Xpa-WT and Xpa-Null HUFs.

Xpa status	Treatment^a^	Clone Id^b^	Codon #	Exon	Mutation type	Strand	WT codon	MUT codon	Coding change	CpG	Zygosity	Contact, structure, zinc	Activity^c^
*TP53*-mutated immortalised clones from HUFs treated with 1 μM 3-NBA													
WT	2	XW-3N-9	120	4	A:T > T:A	NTS	AAG	ATG	K120M		Homo-/hemi-	C	NF
WT	1	XW-3N-59	158	5	G:C > T:A	NTS	CGC	CTC	R158L	CpG	Homo-/hemi-		NF
WT	2	XW-3N-14	161	5	G:C > C:G	NTS	GCC	CCC	A161P		Hetero-		PF
WT	2	XW-3N-15	161	5	G:C > C:G	TS	GCC	GGC	A161G		Hetero-		PF
WT	1	XW-3N-55	175	5	G:C > T:A	NTS	CGC	CTC	R175L	CpG	Homo-/hemi-	S	PF
WT	1	XW-3N-54	178	5	G:C > T:A	TS	CAC	CAA	H178Q		Hetero-		NF
WT	2	XW-3N-8	179	5	G:C > T:A	TS	CAT	AAT	H179N		Homo-/hemi-	Z	PF
WT	1	XW-3N-54	179	5	A:T > C:G	NTS	CAT	CCT	H179P		Hetero-	Z	PF
WT	2	XW-3N-15	194	6	A:T > C:G	TS	CTT	CGT	L194R		Hetero-		NF
WT	2	XW-3N-7	236	7	A:T > G:C	NTS	TAC	TGC	Y236C		Homo-/hemi-		NF
WT	1	XW-3N-43	248	7	G:C > A:T	TS	CGG	TGG	R248W	CpG	Hetero-	C	NF
WT	2	XW-3N-14	249	7	G:C > C:G	NTS	AGG	AGC	R249S		Hetero-	S	NF
WT	1	XW-3N-37	282	8	G:C > A:T	TS	CGG	TGG	R282W	CpG	Homo-/hemi-	S	NF
WT	1	XW-3N-29	in. 3 (SA)		G:C > A:T	NTS					Homo-/hemi-		N/A
Null	1	XN-3N-151	124	4	insertion, G	NTS	TGC	TG-G	Frameshift		Homo-/hemi-		N/A
Null	2	XN-3N-117	157	5	G:C > T:A	NTS	GTC	TTC	V157F	CpG	Hetero-		NF
Null	2	XN-3N-120	157	5	G:C > T:A	NTS	GTC	TTC	V157F	CpG	Hetero-		NF
Null	1	XN-3N-137	244	7	G:C > C:G	NTS	GGC	GCC	G244A		Hetero-		NF
Null	1	XN-3N-136	245	7	G:C > C:G	NTS	GGC	CGC	G245R	CpG	Homo-/hemi-	S	NF
Null	1	XN-3N-137	249	7	G:C > C:G	NTS	AGG	ACG	R249T		Hetero-	S	NF
Null	1	XN-3N-156	273	8	G:C > T:A	TS	CGT	AGT	R273S	CpG	Hetero-	C	NF
Null	2	XN-3N-105	273	8	G:C > T:A	NTS	CGT	CTT	R273L	CpG	Hetero-	C	NF
Null	1	XN-3N-140	277	8	G:C > T:A	NTS	TGT	TTT	C277F		Hetero-	C	NF
Null	1	XN-3N-141	277	8	G:C > A:T	NTS	TGT	TAT	C277Y		Hetero-	C	NF
Null	2	XN-3N-110	in. 5 (SA)		G:C > A:T	NTS			splice		Homo-/hemi-		N/A
Null	1	XN-3N-157	in. 8 (SA)		G:C > T:A	NTS			splice		Homo-/hemi-		N/A
*TP53*-mutated spontaneously immortalised clones from control HUFs (treated with 0.1% DMSO)^d^													
WT		XW-C-115	138	5	G:C > C:G	NTS	GCC	CCC	A138P		Homo-/hemi-		NF
WT		XW-C-137	173	5	A:T > C:G	TS	GTG	GGG	V173G		Homo-/hemi-		NF
Null		XN-C-325	113	4	A:T > C:G	TS	TTC	GTC	F113V		Homo-/hemi-		NF
Null		XN-C-338	113	4	A:T > C:G	TS	TTC	GTC	F113V		Hetero-		NF

a Treatment refers to 1 or 2 × 48 h.

b XW = Xpa-WT; XN = Xpa-Null; 3N = 3-NBA- treated; C = control.

c The overall transactivation activity of the mutant in a yeast functional assay published by Kato et al. [46]. (NF = non-functional, PF = partially functional, F = functional, NA = not assessed).

d As published previously [27].
