# Supplementary material for: TP53 and lacZ mutagenesis induced by 3-nitrobenzanthrone in Xpa-deficient human TP53 knock-in mouse embryo fibroblasts
Source: DNA Repair (Amst). 2016 Mar;39:21–33. doi: 10.1016/j.dnarep.2015.11.004 (PMC4798848; doi:10.1016/j.dnarep.2015.11.004)
Supplement: Supplementary file 1 [file mmc1.docx]

**Supplementary Tables and Figures**

**Supplementary Table 1. Primer sequences and PCR conditions to genotype for *Hupki* and mouse *Trp53* alleles, WT and knockout *Xpa* alleles and the *lacZ* reporter gene.**

**Supplementary Table 2. Primer sequences, PCR conditions and reference sequences for sequencing exons 4–9 of Hupki *TP53*.** In (C): intronic sequence is lower case, exonic sequence is upper case, and primer binding sites are bold/underlined.

**Supplementary Table 3. Sequence context of *TP53* mutations induced by 3-NBA in HUFs.** For each type of single base substitution or insertion, the 5’ and 3’ base are shown. The presence of a mutation at a methylated CpG site is indicated by ‘CpG’. ‘DNA strand’ indicates the strand of the G or A substitution. SA = splice acceptor site; NTS = non-transcribed strand; TS = transcribed strand.

**Supplementary Table 4. The occurrence in human cancer of *TP53* mutations found in Xpa-WT and Xpa-Null HUFs treated with 3-NBA.**

**Supplementary Table 5. *TP53* mutations induced by 3-NBA in the current study compared with mutations induced in previous HIMAs.** Codons mutated in previous HIMAs [3-10] and exact mutation matches are indicated with an ‘x’. The number of HUF cell lines in which a mutation previously occurred is shown.

**Supplementary Table 5 continued.**

**Supplementary Table 5 continued.**

**Supplementary Fig. 1. Bioactivation of 3-NBA and structure of DNA adducts.** NQO1, NAD(P)H:quinone oxidoreductase; XO, xanthine oxidase; POR, NADPH:P450 oxidoreductase; CYP, cytochrome P450; NAT, *N*-acetyltransferase; SULT, sulfotransferase; dA-*N*^6^-3-ABA, 2-(2’-deoxyadenosine-*N*^6^-yl)-3-aminobenzanthrone; dG-*N*^2^-3-ABA, 2-(2’-deoxyguanosin-*N*^2^-yl)-3-aminobenzanthrone; dG-C8-*N*-3-ABA, *N*-(2’-deoxyguanosin-8-yl)-3-aminobenzanthrone. Adapted from Arlt *et al.* [11].

**Supplementary Fig. 2. Pattern of DNA adducts induced in Xpa-WT and Xpa-Null Hupki mice treated with 3-NBA.** Mice were treated with (A) 2 or (B) 0.2 mg/kg bw 3-NBA. Autoradiographic profiles in different tissues were detected by ^32^P-postlabelling; the origins, in the bottom left-hand corner, were cut off before exposure. Spot 1: 2-(2'-deoxyadenosin-*N*^6^-yl)-3-aminobenzanthrone (dA-*N*^6^-3-ABA), Spot 3: 2-(2'-deoxyguanosin-*N*^2^-yl)-3-aminobenzanthrone (dG-*N*^2^-3-ABA), Spot 4: *N*-(2'-deoxyguanosin-8-yl)-3-aminobenzanthrone (dG-C8-*N*-3-ABA). Spot 2 is a deoxyadenosine adduct that has not yet been structurally characterised. Solvent conditions for the resolution of ^32^P-labelled adducts on polyethyleneimine-cellulose (PEI) thin-layer chromatography (TLC) were: D1: 1.0 M sodium phosphate (pH 6.0); D2: 4.0 M lithium formate, 7.0 M urea (pH 3.5); D3: 0.8 M lithium chloride, 0.5 M Tris-HCl, 8.5 M urea (pH 8.0) [11, 12].

**Supplementary Fig. 3. Levels of DNA adducts in Hupki mice 5 days after a single treatment with 3-NBA.** Xpa-WT and Xpa-Null mice were treated with a single dose of 3-NBA (2 mg/kg bw). DNA adduct levels (RAL, relative adduct labelling) in different tissues were assessed 5 days later by ^32^P-postlabelling. Values represent means ± SD from 3 animals and each DNA sample was measured by two independent ^32^P-postlabelling analyses. † = not determined due to death of Xpa-Null animals.

**Supplementary Fig. 4. Stabilisation and activation of p53 in Xpa-WT and Xpa-Null HUFs treated with 3-NBA.** Protein expression of p53, phosphorylated p53 (Ser15) and p21 was assessed by Western blotting using whole cell lysates of Xpa-WT and Xpa-Null HUFs treated with 0.25–1.00 µM 3-NBA for 48 hr. Gapdh expression is shown as a loading control. HUFs treated with 0.25 µM BaP were included for comparison.

**References for Supplementary Material**

[1] D.M. DeMarini, S. Landi, D. Tian, N.M. Hanley, X. Li, F. Hu, B.C. Roop, M.J. Mass, P. Keohavong, W. Gao, M. Olivier, P. Hainaut, J.L. Mumford, Lung tumor KRAS and TP53 mutations in nonsmokers reflect exposure to PAH-rich coal combustion emissions, Cancer Res, 61 (2001) 6679-6681.

[2] F.H. Sarkar, Y. Li, V. Vallyathan, Molecular analysis of p53 and K-ras in lung carcinomas of coal miners, International journal of molecular medicine, 8 (2001) 453-459.

[3] J. vom Brocke, A. Krais, C. Whibley, M.C. Hollstein, H.H. Schmeiser, The carcinogenic air pollutant 3-nitrobenzanthrone induces GC to TA transversion mutations in human p53 sequences, Mutagenesis, 24 (2009) 17-23.

[4] J.E. Kucab, H. van Steeg, M. Luijten, H.H. Schmeiser, P.A. White, D.H. Phillips, V.M. Arlt, TP53 mutations induced by BPDE in Xpa-WT and Xpa-Null human TP53 knock-in (Hupki) mouse embryo fibroblasts, Mutation Research/Fundamental and Molecular Mechanisms of Mutagenesis, 773 (2015) 48-62.

[5] Z. Liu, K.R. Muehlbauer, H.H. Schmeiser, M. Hergenhahn, D. Belharazem, M.C. Hollstein, p53 mutations in benzo(a)pyrene-exposed human p53 knock-in murine fibroblasts correlate with p53 mutations in human lung tumors, Cancer Res, 65 (2005) 2583-2587.

[6] M. Reinbold, J.L. Luo, T. Nedelko, B. Jerchow, M.E. Murphy, C. Whibley, Q. Wei, M. Hollstein, Common tumour p53 mutations in immortalized cells from Hupki mice heterozygous at codon 72, Oncogene, 27 (2008) 2788-2794.

[7] T. Nedelko, V.M. Arlt, D.H. Phillips, M. Hollstein, TP53 mutation signature supports involvement of aristolochic acid in the aetiology of endemic nephropathy-associated tumours, Int J Cancer, 124 (2009) 987-990.

[8] N. Feldmeyer, H.H. Schmeiser, K.R. Muehlbauer, D. Belharazem, Y. Knyazev, T. Nedelko, M. Hollstein, Further studies with a cell immortalization assay to investigate the mutation signature of aristolochic acid in human p53 sequences, Mutat Res, 608 (2006) 163-168.

[9] Z. Liu, M. Hergenhahn, H.H. Schmeiser, G.N. Wogan, A. Hong, M. Hollstein, Human tumor p53 mutations are selected for in mouse embryonic fibroblasts harboring a humanized p53 gene, Proc Natl Acad Sci U S A, 101 (2004) 2963-2968.

[10] C. Whibley, A.F. Odell, T. Nedelko, G. Balaburski, M. Murphy, Z. Liu, L. Stevens, J.H. Walker, M. Routledge, M. Hollstein, Wild-type and Hupki (human p53 knock-in) murine embryonic fibroblasts: p53/ARF pathway disruption in spontaneous escape from senescence, J Biol Chem, 285 (2010) 11326-11335.

[11] V.M. Arlt, H.H. Schmeiser, M.R. Osborne, M. Kawanishi, T. Kanno, T. Yagi, D.H. Phillips, T. Takamura-Enya, Identification of three major DNA adducts formed by the carcinogenic air pollutant 3-nitrobenzanthrone in rat lung at the C8 and N2 position of guanine and at the N6 position of adenine, Int J Cancer, 118 (2006) 2139-2146.

[12] V.M. Arlt, C.A. Bieler, W. Mier, M. Wiessler, H.H. Schmeiser, DNA adduct formation by the ubiquitous environmental contaminant 3-nitrobenzanthrone in rats determined by (32)P-postlabeling, Int J Cancer, 93 (2001) 450-454.
